# Hertfordshire Partnership NHS Trust - Improving VTE Risk Assessments in an Older Adult Psychiatric Inpatient Unit

**DOI:** 10.1192/bjo.2022.313

**Published:** 2022-06-20

**Authors:** Bhavna Khanna, Christina O'Dowd

**Affiliations:** Hertfordshire Partnership University NHS Foundation Trust, St Albans, United Kingdom

## Abstract

**Aims:**

VTE-related deaths are a leading cause of preventable mortality amongst all hospital inpatients. Psychiatric inpatients are at greater risk of this, due to administration of antipsychotic medication and longer inpatient stay. This is particularly significant during the COVID-19 pandemic, not least as it is a disease known to cause hypercoagulability, but also due to an increase in mental illness and extended admission, resulting from an overwhelmingly run social service. The objective is to analyse VTE risk assessments performed for the 23 patients at Lambourn Grove, a continuing care unit for old age psychiatric inpatients, diagnosed with dementia. The aims are to assess; the frequency at which VTE risk assessments are performed, the accuracy of each assessment and the subsequent management and appropriateness of preventative measures taken.

**Methods:**

A retrospective study was conducted of 23 service users. Data were collected from the VTE assessment form on the local electronic patient record system, and analysed to assess compliance with local guidance. GP records were consulted to cross check data and ensure accuracy of information inputted. Improvement measures include presenting at the local teaching session and implementing a mandatory monthly review onto the ward round proforma. A second cycle will be carried out to assess the success of these interventions in improving best medical practice.

**Results:**

Of the 23 service users, 20 patients had their VTE risk assessment completed on admission and there was a delay of over a month with the remaining 3 patients. Of significance is that of all initial VTEs, 6 out of 23 contained inaccurate details, such as omission of comorbidities or a subjective assessment of mobility, indicating the need to use a standardized tool which allows for comparison across time. The mean admission duration for all 23 inpatients, as of February 2022 was calculated to be 16.2 months, with a range of 2 and 59 months. 15 patients did not have their VTE risk assessment repeated during admission, and of these 2 did have a change in their risk profile, indicating non-adherence with NICE guidelines.

**Conclusion:**

This study has identified significant areas for improvement, specifically the need for clear timing for repeated VTE assessments, consistent sources of patient's medical history and documentation of mobility status. The project has highlighted the need for a more robust VTE assessment protocol which is currently being developed, to improve patient mortality and outcomes.

